# Supplementary material for: OTTERS: a powerful TWAS framework leveraging summary-level reference data
Source: Nat Commun. 2023 Mar 7;14:1271. doi: 10.1038/s41467-023-36862-w (PMC9992663; doi:10.1038/s41467-023-36862-w)
Supplement: Supplementary file 1 — Supplementary Information [file 41467_2023_36862_MOESM1_ESM.pdf]

Supplementary Information

1. Supplementary Figures

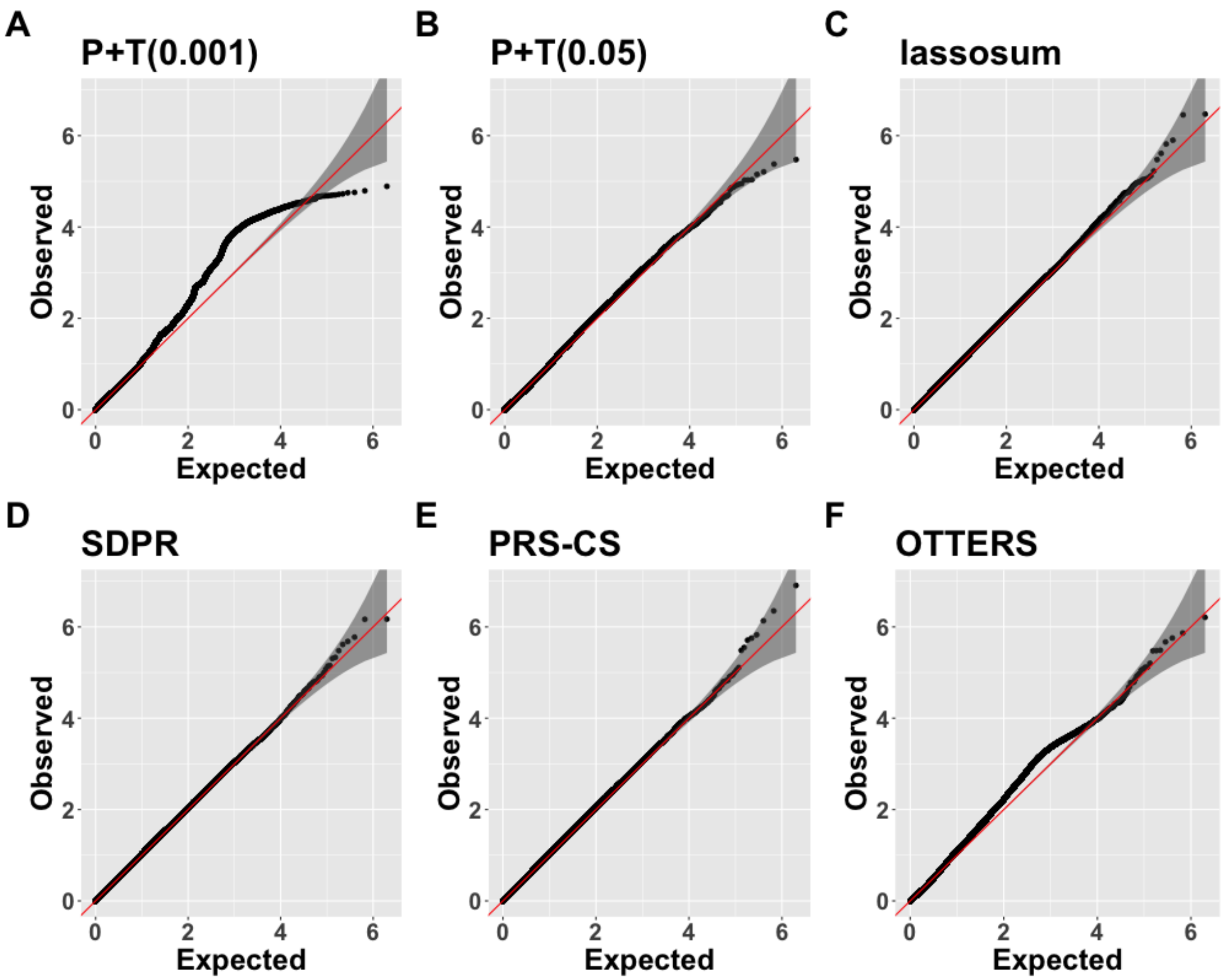

Figure S1. Quantile-Quantile (QQ) plots of TWAS p-values for P+T(0.001), P+T(0.05), lassosum, SDPR, PRS-CS, and OTTERS under 10^6 null simulations.
Phenotype data were simulated from $N(0, 1)$. Gene expression were generated with $h_e^2 = 0.1$ and $p_{causal} = 0.001$. eQTL weights were permutated 2×10^3 times for 500 genes to perform 10^6 null simulations of TWAS. P-values were the raw p-values from TWAS Z-score tests (two-sided). Error bands denote the 95% confidence interval of the distribution of p-values under null hypothesis (Expected).

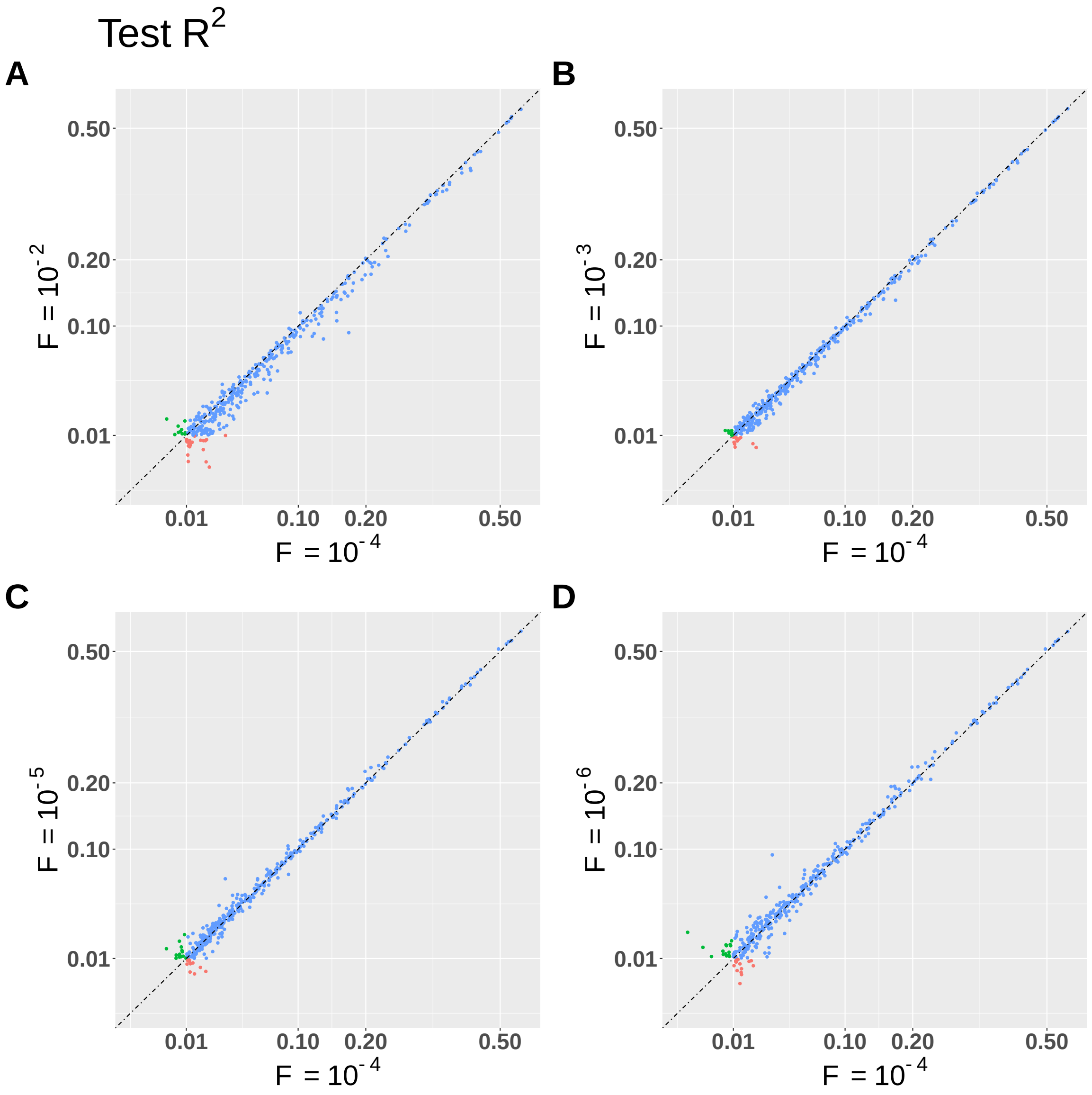

Figure S2. Test R^2 in GTEx V8 samples by PRS-CS with $\phi = (10^{-2}, 10^{-3}, 10^{-5}, 10^{-6})$ vs. $\phi = 10^{-4}$ for 590 genes on chromosome 4.

Different colors denote whether the gene with test $\mathrm{R}^2 > 0.01$ was obtained by using both ϕ values (blue), or only by using one of the compared ϕ values (red for the ϕ on the x axis and green for the ϕ on the y axis). Genes with training $\mathrm{R}^2 \leq 0.01$ by both methods were not shown in the plot.

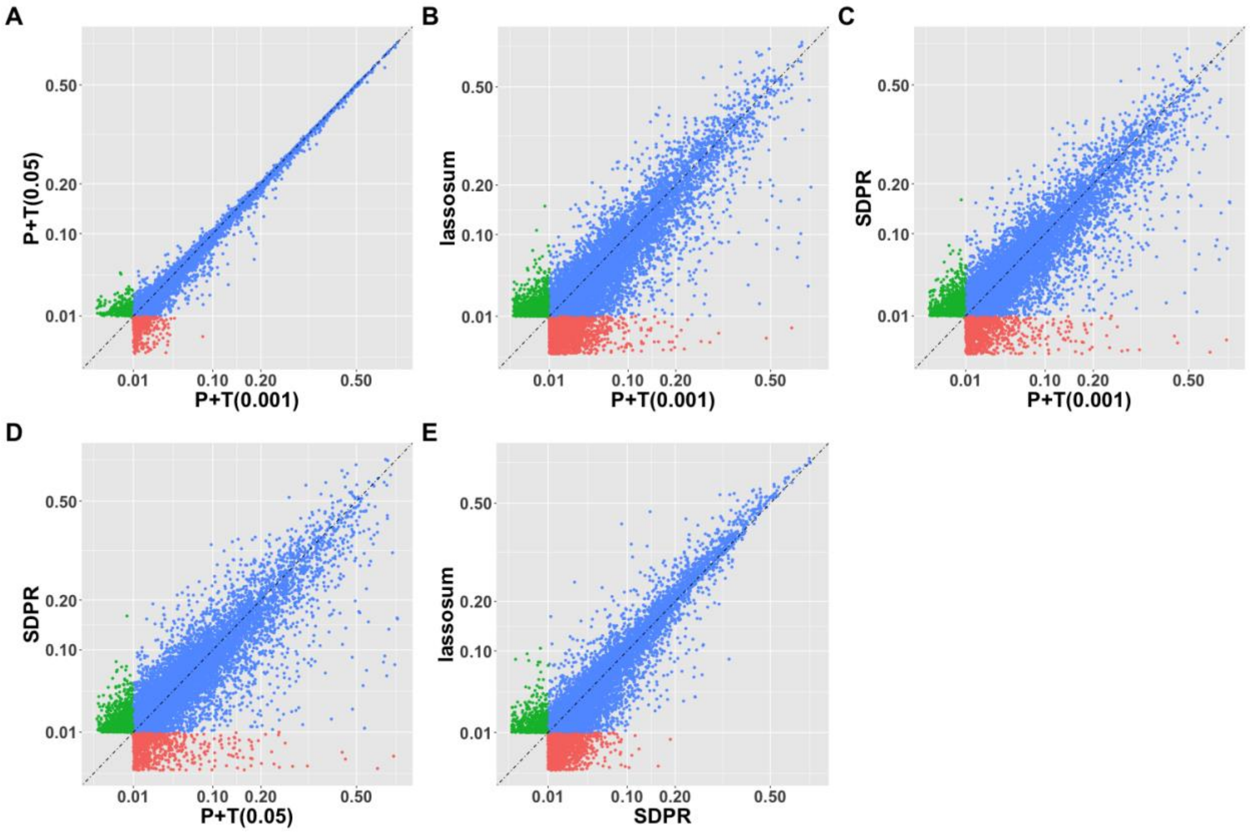

Figure S3. Test R^2 in GTEx V8 whole blood samples by P+T(0.001), P+T(0.05), lassosum, and SDPR.

Different colors denoting whether the gene with test R^2> 0.01 was obtained by both methods (blue), or only by one of the pair-wise compared methods (red for the method on x axis and green for the method on y axis).

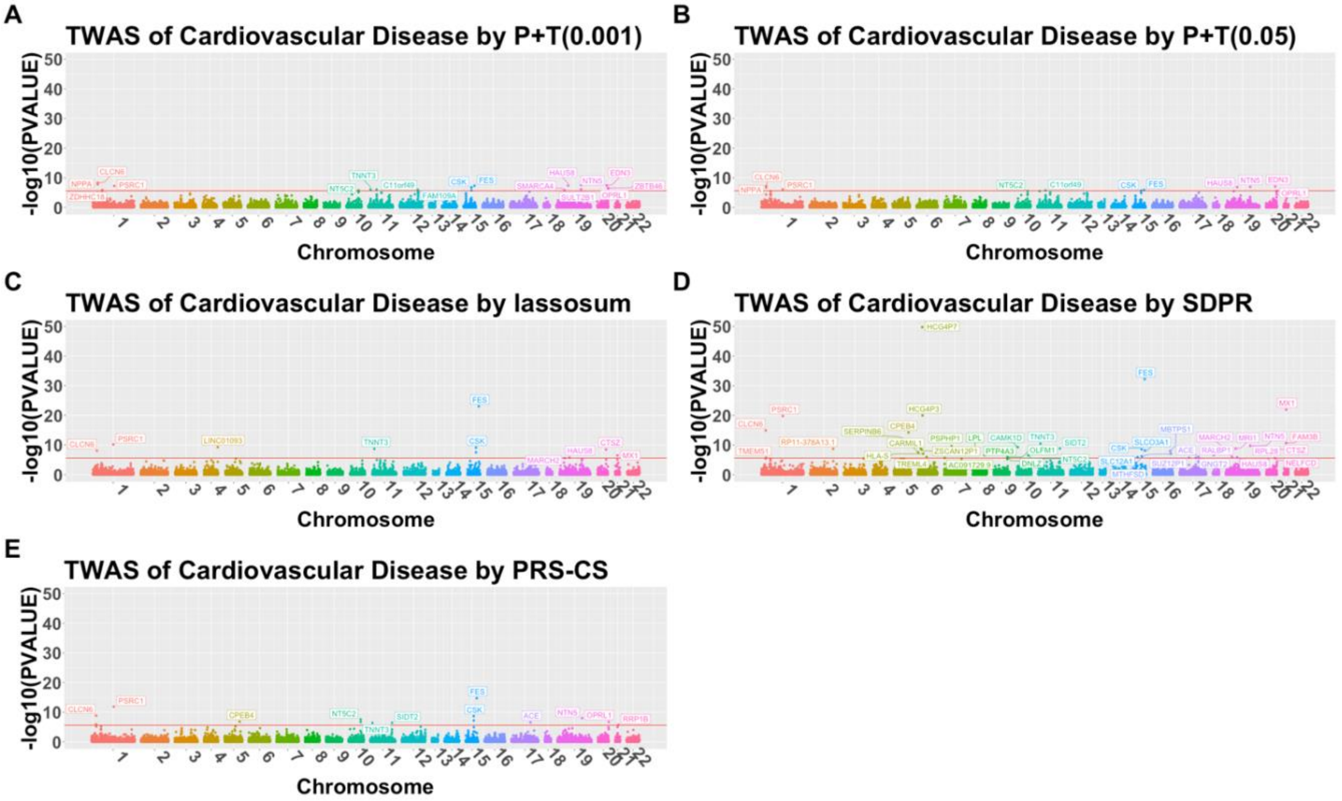

Figure S4. Manhattan plots of TWAS of cardiovascular disease by P+T(0.001) (A), P+T(0.05) (B), lassosum (C), SDPR (D), and PRS-CS (E) with independently significant TWAS risk genes being labelled.
The x-axis represented the genomic position, and the y-axis represented $-\log_{10}$(p-values). P-values were the genomic-control corrected p-values from the TWAS Z-score test (two-sided).

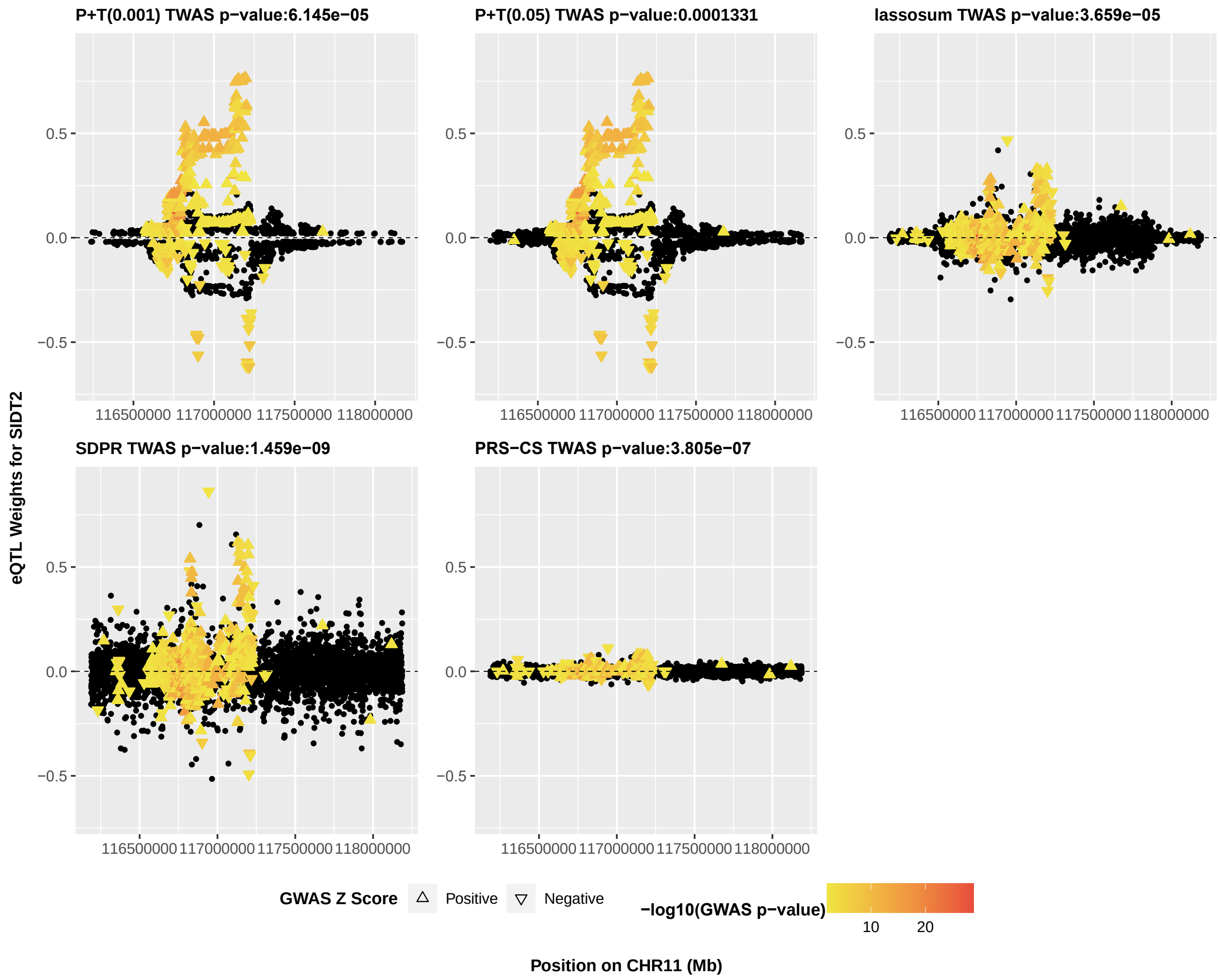

Figure S5. Cis-eQTL weights estimated by individual methods for gene *SIDT2*. Color coded with respect to $-\log_{10}$(p-value) from the UKBB GWAS summary statistics and shape coded with respect to the direction of GWAS Z-score test statistics. TWAS P-values shown in figure titles were the genomic-control corrected from TWAS Z-score tests (two-sided).

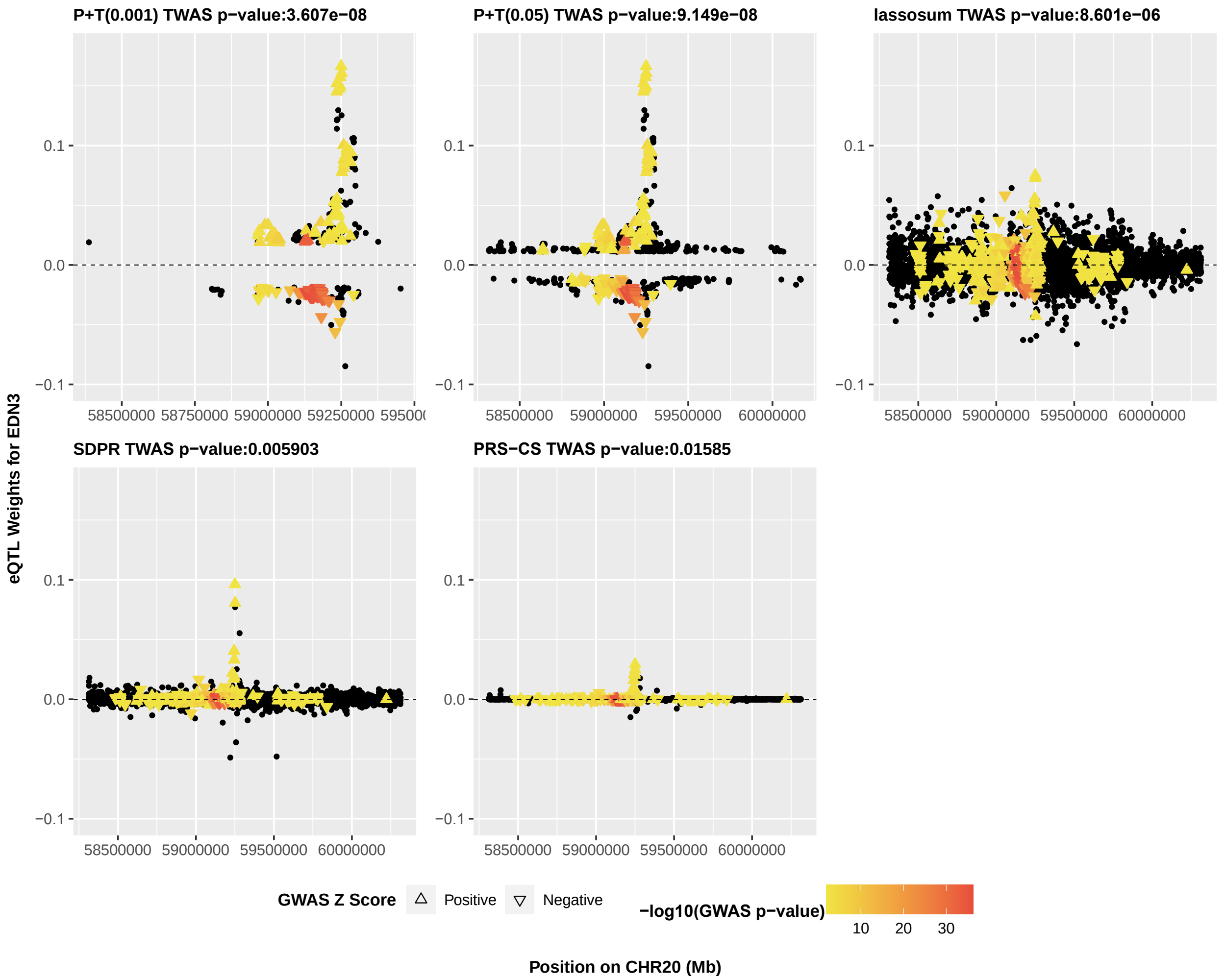

Figure S6. Cis-eQTL weights estimated by individual methods for gene *EDN3*. Color coded with respect to $-\log_{10}$(p-value) from the UKBB GWAS summary statistics and shape coded with respect to the direction of GWAS Z-score statistics. TWAS P-values shown in figure titles were the genomic-control corrected p-values from TWAS Z-score tests (two-sided).

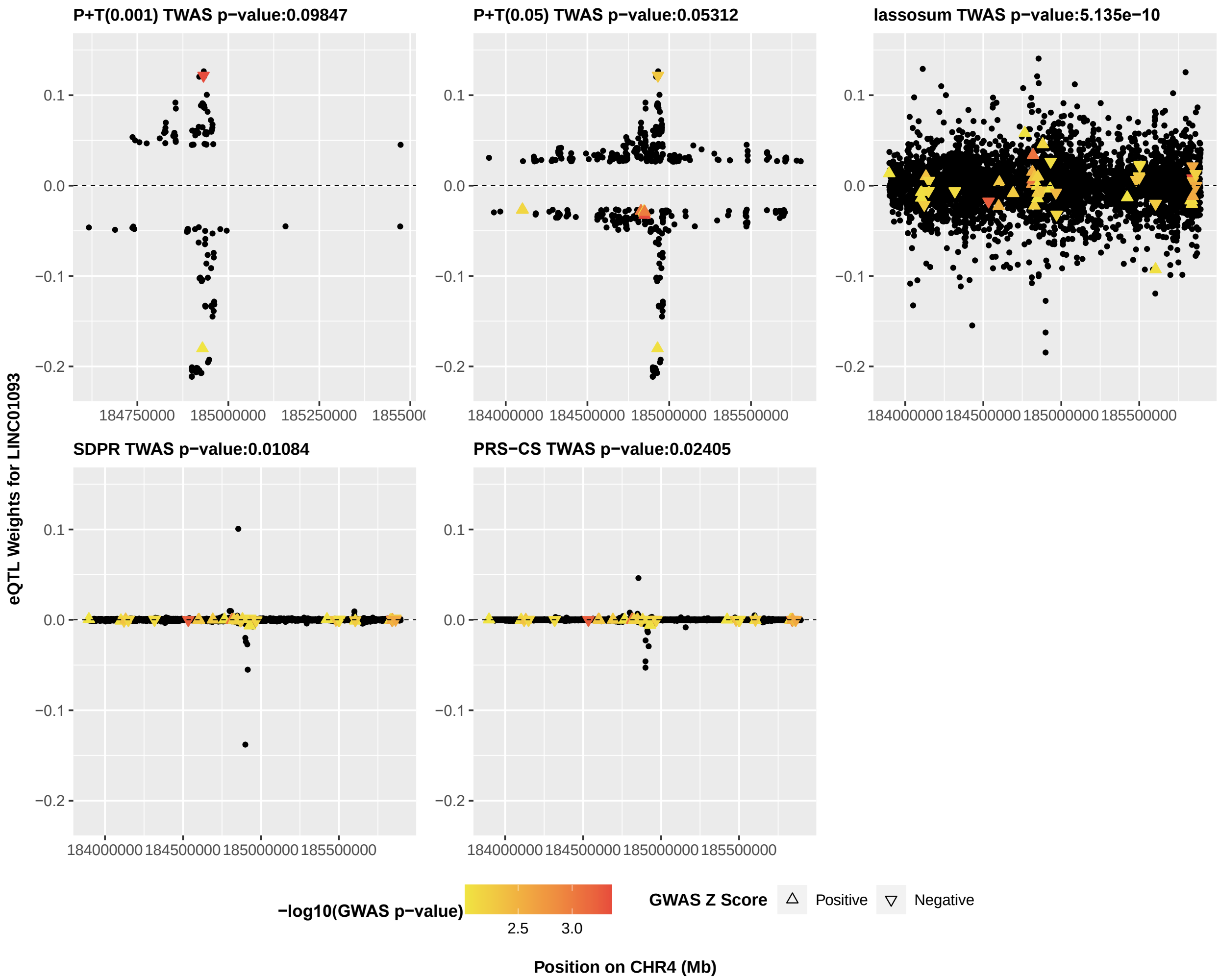

Figure S7. cis-eQTL weights estimated by individual methods for gene *LINC01093*. Color coded with respect to $-\log_{10}$(p-value) from the UKBB GWAS summary statistics and shape coded with respect to the direction of GWAS Z-score statistics. TWAS p-values shown in figure titles were the genomic-control corrected p-values from TWAS Z-score tests (two-sided).

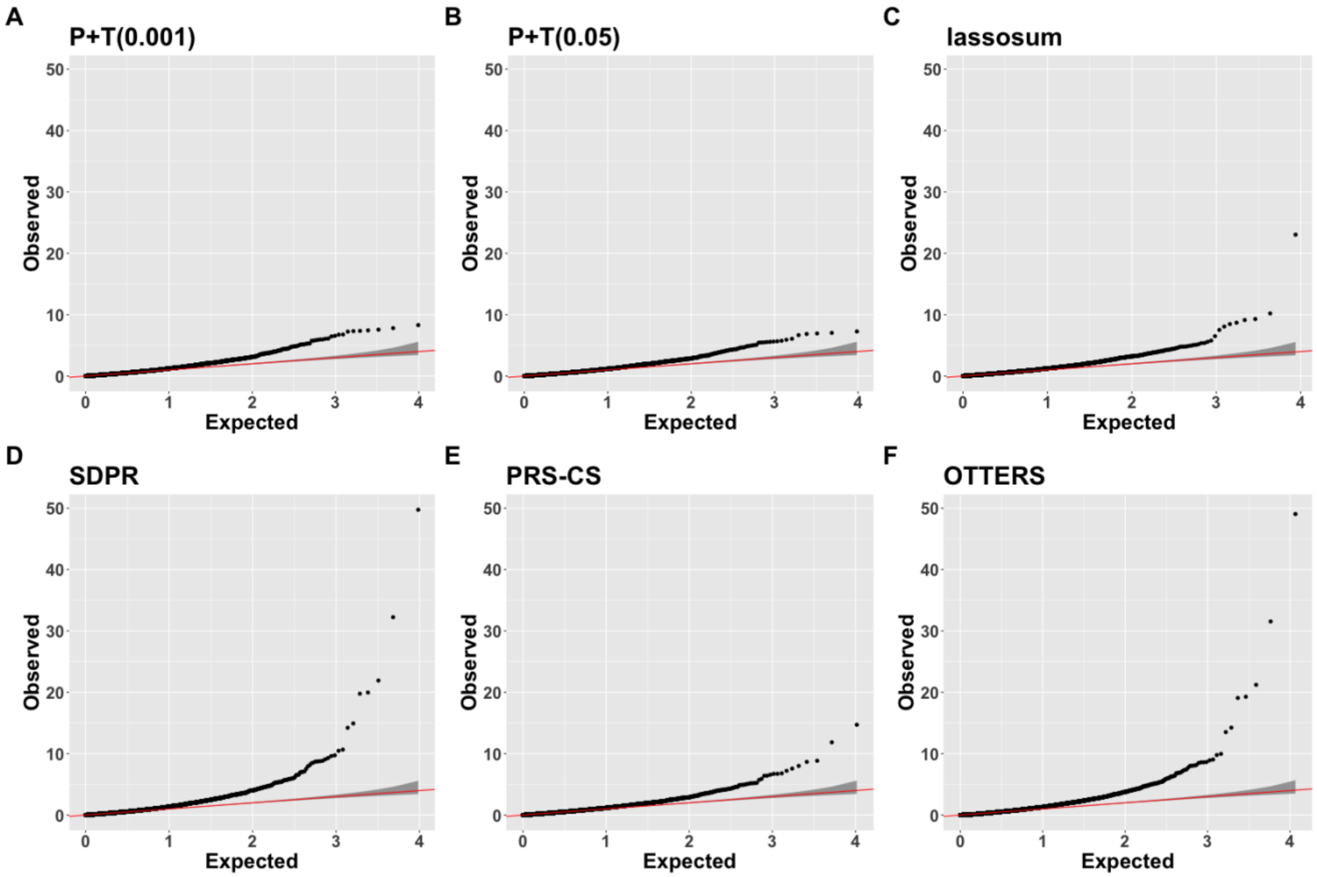

Figure S8. Quantile-Quantile plots of TWAS p-values of cardiovascular disease of 9,827 genes by P+T(0.001) (A), 9,677 genes by P+T(0.05) (B), 8,695 genes by lassosum (C), 9,641 genes by SDPR (D), 10,380 genes by PRS-CS (E), and 11,641 genes by OTTERS (F). Genes with test GReX $R^2 > 0.01$ were included in the plot. P-values were the genomic-control corrected p-values from TWAS Z-score tests (two-sided). Error bands denote the 95% confidence interval of the distribution of p-values under null hypothesis (Expected).

A.

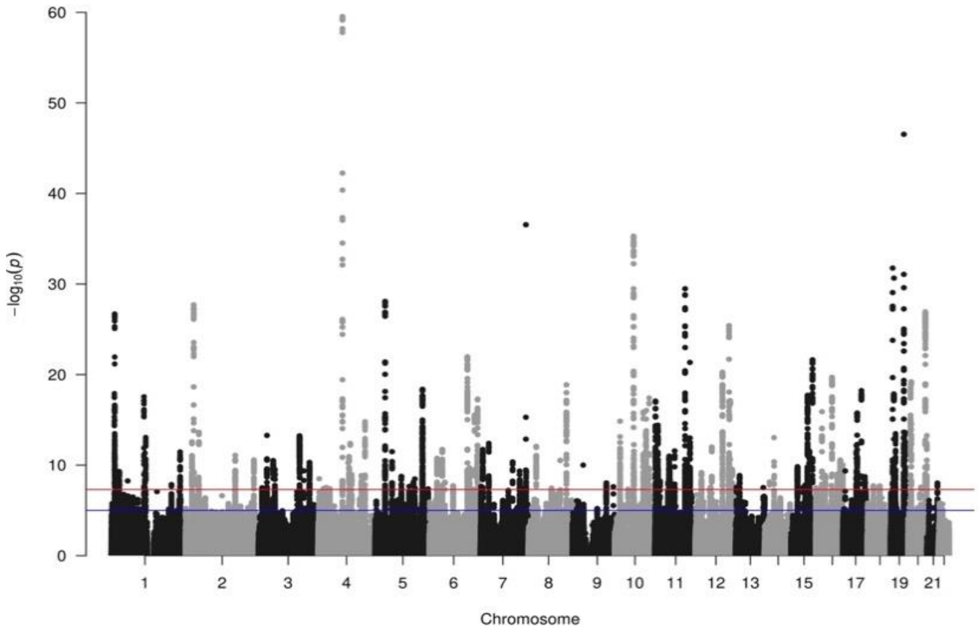

B.

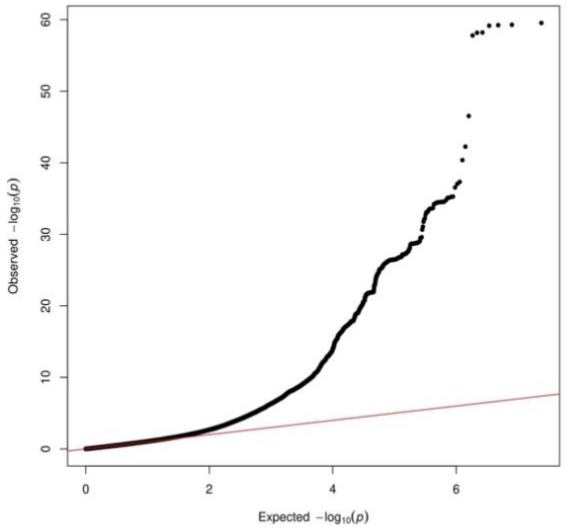

Figure S9. Manhattan Plot (A) and Quantile-Quantile plot (B) of GWAS p-values from UK Biobank GWAS summary results of cardiovascular disease.

P-values were the genomic-control corrected p-values from GWAS Z-score tests (two-sided).

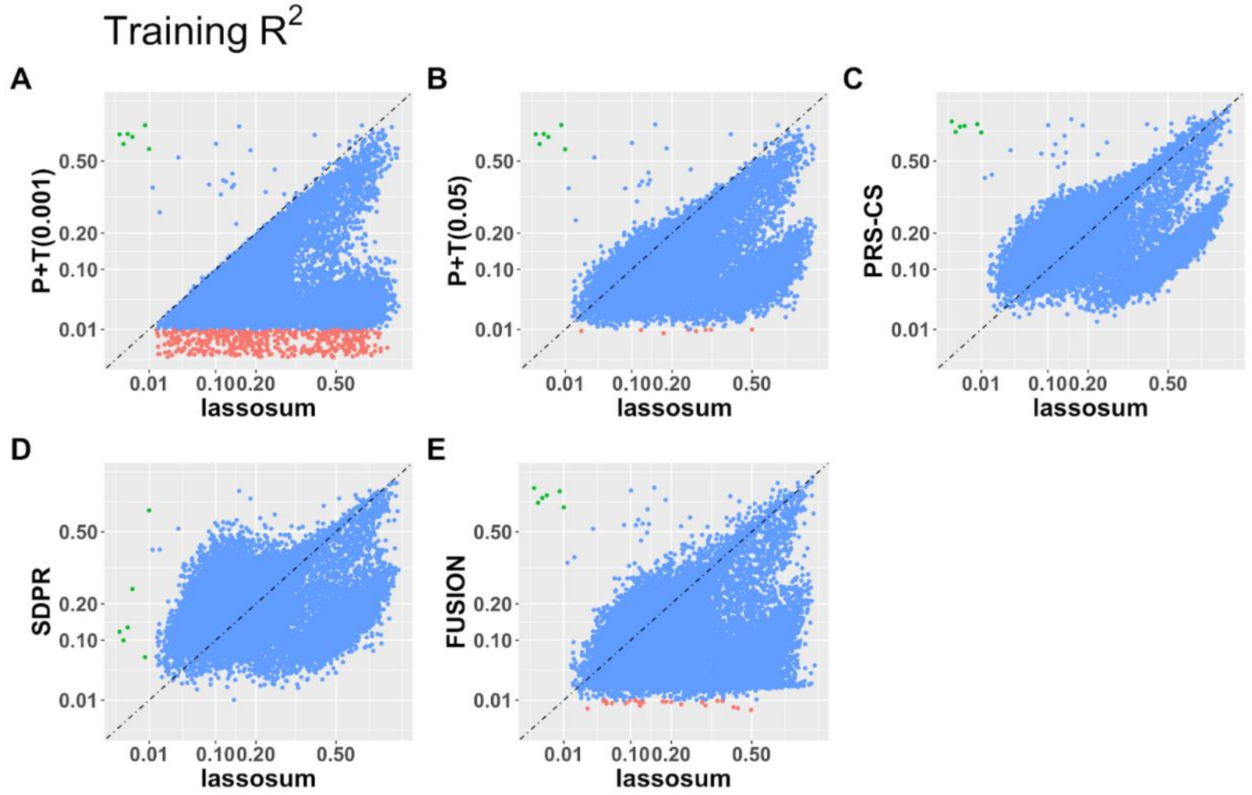

Figure S10. Training R^2 in GTEx V8 whole blood samples by lassosum versus P+T(0.001), P+T(0.05), PRS-CS, SDPR, and FUSION.

Training R^2 by lassosum versus P+T(0.001) (A), P+T(0.05) (B), PRS-CS (C), SDPR (D), and FUSION (E) with n=574 GTEx V8 training samples, with different colors denoting whether the imputation $R^2 > 0.01$ only by lassosum (red), only by the y axis method (green), or both methods (blue). Genes with $R^2 \leq 0.01$ by both methods were excluded from the plot.

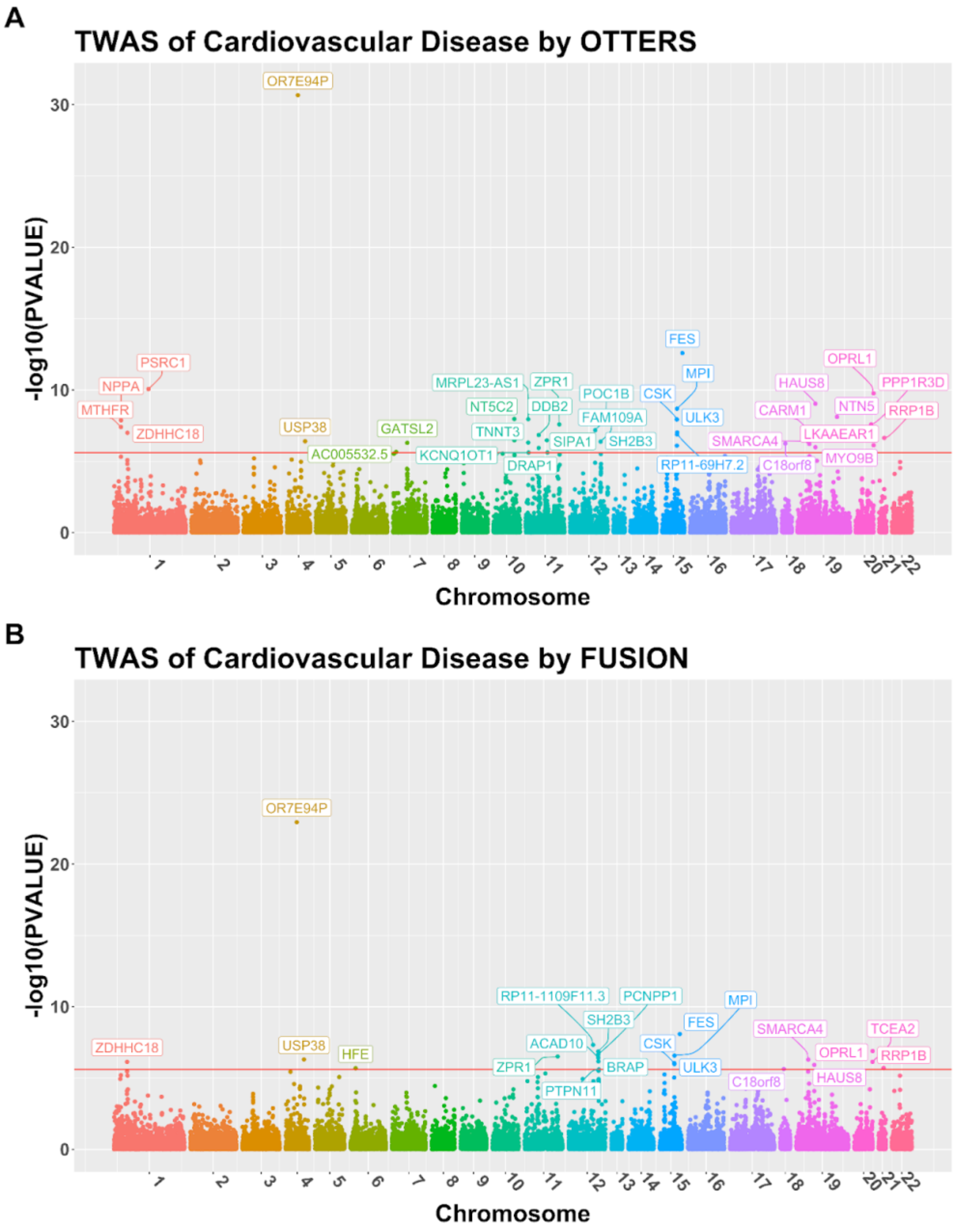

Figure S11. Manhattan plot of TWAS p-values by OTTERS (A) and FUSION (B) using eQTL data from GTEx V8 whole blood samples.

The x-axis represented the genomic position, and the y-axis represented $-\log_{10}$(p-values). P-values were the genomic-control corrected p-values from TWAS Z-score tests (two-sided). Independently significant TWAS risk genes were labeled.

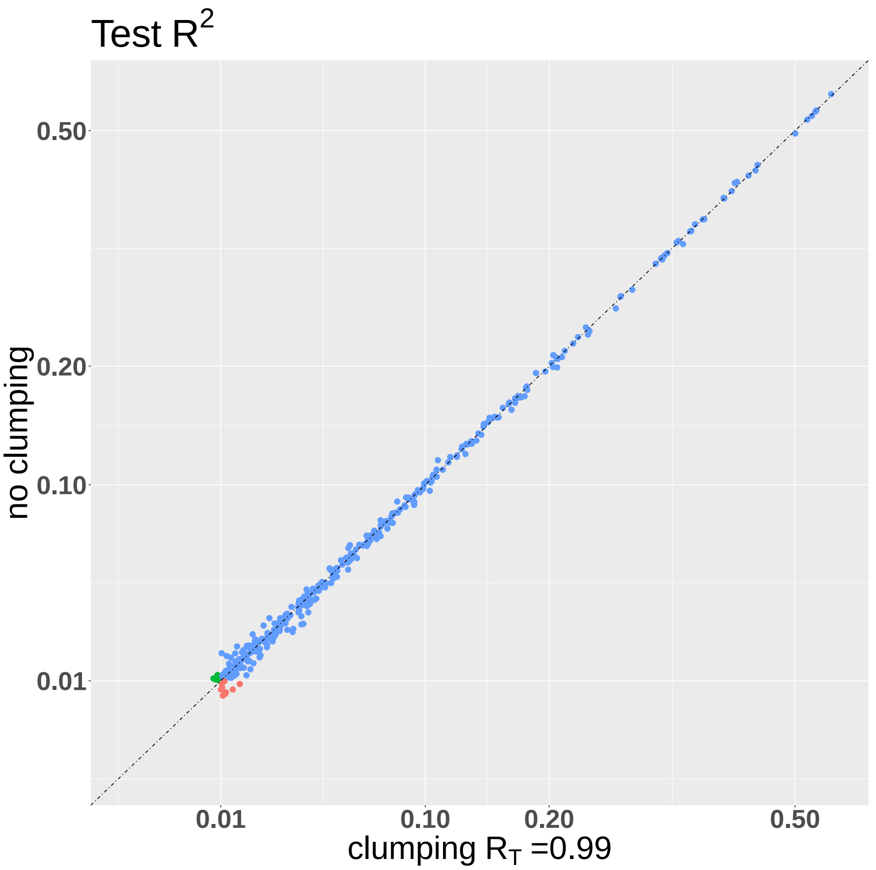

Figure S12. Test R^2 in GTEx V8 samples by PRS-CS with LD-clumping (R_T = 0.99) versus no clumping.

Test R^2 in n=315 GTEx V8 test samples by PRS-CS with LD-clumping (R_T = 0.99) versus no clumping, with different colors denoting whether the imputation R^2 > 0.01 only by clumping (red), only by no clumping (green), or both (blue). Genes with $R^2 \leq 0.01$ by both methods were excluded from the plot.

2. Supplementary Tables

Table S1. TWAS type I errors under 10^6 null simulation studies with $\mathbf{h_e^2 = 0.1}$ and $\mathbf{p_{causal} = 0.001}$ by 5 individual methods P+T(0.001), P+T(0.05), lassosum, SDPR, PRS-CS, and OTTERS.

Significance Level	P+T(0.001)	P+T(0.05)	lassosum	SDPR	PRS-CS	OTTERS
$\mathbf{1.0 \times 10^{-2}}$	1.71×10^{-2}	1.25×10^{-2}	1.12×10^{-2}	1.08×10^{-2}	1.12×10^{-2}	1.60×10^{-2}
$\mathbf{1.0 \times 10^{-4}}$	7.13×10^{-5}	9.60×10^{-5}	1.32×10^{-4}	9.70×10^{-5}	1.13×10^{-4}	9.30×10^{-5}
$\mathbf{2.5 \times 10^{-6}}$	0	0	5.00×10^{-6}	5.00×10^{-6}	5.00×10^{-6}	4.00×10^{-6}

Table S2. Independent significant TWAS genes of cardiovascular disease identified by individual training methods (P+T(0.001), P+T(0.05), lassosum, SDPR, PRS-CS) but not by OTTERS. P-values were the genomic-control corrected p-values from TWAS Z-score tests (two-sided).

	CHR	GENE	P-VALUE	OTTERS P-VALUE
**P+T (0.001)**	1	ZDHHC18	1.2602E-06	3.8725E-06
	12	FAM109A	1.1125E-06	3.8897E-06
	19	SMARCA4	1.4672E-06	5.0821E-06
**lassosum**	19	MARCH2	2.6164E-06	4.0247E-06
**SDPR**	1	TMEM51	1.8037E-06	9.0175E-06
	6	TREML4	9.6488E-07	4.8197E-06
	7	AC091729.9	2.3835E-06	1.191E-05
	9	OLFM1	1.3473E-06	6.736E-06
	9	DNLZ	1.8569E-06	9.2841E-06
	15	SLC12A1	1.2396E-06	6.1852E-06
	17	SUZ12P1	1.9124E-06	9.4828E-06
	17	GNGT2	2.0687E-06	9.8317E-06
	19	MARCH2	1.1691E-06	4.0247E-06
	20	NELFCD	2.8779E-06	1.4186E-05
**PRS-CS**	21	RRP1B	1.8223E-06	6.9344E-06

Table S3. Independent TWAS risk genes of cardiovascular disease identified by OTTERS using eQTL summary statistics from GTEx V8 whole blood samples. TWAS p-values by 5 individual PRS methods and OTTERS are shown with significant p-values in bold. P-values were the genomic-control corrected from TWAS Z-score tests (two-sided).

**CHR**	**ID**	OTTERS	**FUSION**	**P+T(0.001)**	**P+T(0.05)**	**lassosum**	**SDPR**	**PRScs**
1	MTHFR	**3.99E-08**	1.12E-03	**8.05E-09**	**1.11E-06**	7.17E-05	1.06E-03	7.95E-05
1	NPPA	**1.33E-08**	6.35E-06	**1.19E-08**	**3.52E-09**	**1.39E-07**	5.71E-04	5.94E-04
1	ZDHHC18	**1.02E-07**	**7.35E-07**	**1.82E-07**	**5.27E-07**	**2.67E-08**	**9.34E-07**	**3.18E-07**
1	PSRC1	**8.75E-11**	1.70E-04	**1.91E-11**	**1.61E-06**	**1.23E-09**	**3.11E-10**	**1.45E-09**
4	OR7E94P	**2.14E-31**	**1.16E-23**	**4.28E-32**	2.53E-04	**2.16E-10**	2.80E-05	**1.35E-11**
4	USP38	**3.98E-07**	**4.99E-07**	2.85E-05	1.09E-04	**8.12E-08**	7.85E-06	1.41E-05
7	AC005532.5	**2.26E-06**	1.03E-03	**4.56E-07**	4.88E-05	3.89E-04	1.17E-03	7.48E-04
7	GATSL2	**5.12E-07**	1.69E-02	**7.59E-07**	**1.18E-07**	1.70E-03	3.45E-03	7.02E-04
10	NT5C2	**1.09E-08**	2.42E-04	**2.69E-09**	**1.76E-06**	**1.17E-08**	1.03E-03	6.62E-05
11	TNNT3	**4.89E-07**	1.65E-05	**1.01E-07**	1.16E-05	7.80E-06	2.72E-05	3.02E-05
11	MRPL23-AS1	**1.13E-08**	2.48E-01	**2.26E-09**	1.21E-05	8.89E-05	7.11E-02	8.61E-04
11	KCNQ1OT1	**2.47E-06**	7.37E-03	**5.09E-07**	1.32E-04	1.80E-05	1.85E-02	4.39E-03
11	DDB2	**1.46E-07**	2.14E-04	**1.00E-07**	3.52E-06	**4.17E-08**	4.10E-04	1.18E-03
11	SIPA1	**3.42E-07**	4.77E-06	**1.24E-07**	3.49E-05	**1.72E-07**	4.31E-05	**1.45E-06**
11	DRAP1	**2.46E-06**	9.41E-02	**4.92E-07**	4.16E-02	1.88E-01	4.20E-02	9.94E-02
11	ZPR1	**2.64E-08**	**3.11E-07**	**9.47E-07**	**5.34E-09**	2.06E-05	3.44E-06	**1.09E-06**
12	POC1B	**6.76E-08**	8.87E-05	**1.35E-08**	5.55E-05	1.19E-05	9.13E-04	1.08E-04
12	FAM109A	**4.25E-07**	1.44E-05	**3.58E-07**	**3.05E-07**	1.04E-03	**4.69E-07**	**2.82E-07**
12	SH2B3	**4.27E-07**	**1.37E-07**	**2.01E-07**	**5.86E-07**	4.36E-04	**9.32E-07**	**2.52E-07**
15	CSK	**1.18E-08**	**9.12E-07**	**3.12E-07**	**4.20E-07**	5.53E-01	**2.28E-08**	**2.68E-09**
15	ULK3	**1.18E-08**	**1.05E-06**	**8.28E-09**	**1.05E-07**	6.39E-01	**4.27E-09**	**1.68E-08**
15	MPI	**2.08E-09**	**2.69E-07**	**3.61E-08**	**1.58E-07**	4.47E-01	**6.20E-10**	**1.32E-09**
15	RP11-69H7.2	**9.54E-08**	5.12E-03	**1.91E-08**	2.66E-05	1.39E-04	6.35E-05	2.78E-05
15	FES	**2.61E-13**	**8.18E-09**	**8.34E-14**	**1.16E-08**	**4.48E-12**	**4.35E-12**	**1.48E-13**
18	C18orf8	**5.71E-07**	**2.32E-06**	9.16E-06	**1.87E-06**	**5.94E-07**	**1.99E-07**	**7.05E-07**
19	CARM1	**7.43E-08**	7.53E-02	3.12E-06	**1.49E-08**	6.60E-04	2.52E-03	1.87E-03
19	SMARCA4	**6.24E-07**	**5.14E-07**	**4.26E-07**	**9.35E-07**	**2.17E-07**	1.34E-01	1.37E-03
19	HAUS8	**9.29E-10**	**1.20E-06**	**1.56E-09**	**1.85E-09**	**2.38E-10**	**5.11E-07**	**9.97E-08**
19	MYO9B	**1.04E-06**	8.26E-05	**2.15E-07**	9.87E-06	4.28E-04	3.78E-03	2.97E-05
19	NTN5	**7.77E-09**	9.62E-06	**1.55E-09**	3.95E-06	5.29E-06	4.78E-03	2.56E-04
20	PPP1R3D	**2.63E-08**	1.09E-01	3.21E-05	**5.25E-09**	3.51E-01	9.16E-04	8.22E-03
20	OPRL1	**1.74E-10**	**1.28E-07**	**4.16E-08**	**8.25E-07**	**3.53E-11**	**5.25E-09**	**4.55E-09**
20	LKAAEAR1	**7.50E-07**	3.00E-01	**1.50E-07**	8.47E-04	2.68E-01	1.14E-02	1.04E-03
21	RRP1B	**2.35E-07**	**2.00E-06**	**5.73E-08**	**4.56E-07**	**6.27E-07**	8.62E-03	1.32E-04

Table S4. Computational time (minutes) and memory usage (Gigabytes in the parenthesis) of different training methods for genes with different number of test eQTLs after LD-clumping with R_T = 0.99.

For each group with the number of test eQTLs <2000, from 2000 to 3000, from 3000 to 4000, and >4000, the reported computational time and memory usage were the average time and memory per gene used for 10 randomly selected genes on Chromosome 4.

# Test eQTLs[a]	P+T	lassosum	SDPR	PRS-CS
<2000	0.003 (0.0015)	0.16 (0.025)	3.56 (0.024)	4.18 (0.525)
2000 - 3000	0.003 (0.0012)	0.17 (0.027)	9.07 (0.065)	7.68 (0.416)
3000 - 4000	0.005 (0.0013)	0.20 (0.029)	7.96 (0.06)	35.14 (0.573)
>4000	0.005 (0.0008)	0.54 (0.035)	30.89 (0.173)	89.945 (0.577)

a: number of eQTLs per gene after LD-clumping (R_T = 0.99).

3. Supplementary Methods

3.1 OTTERS Stage I with Summary-level Reference Data

OTTERS will train eQTL effect sizes $\hat{\mathbf{w}}$ using the following GReX imputation model (i.e., a multivariate regression model assuming additive genetic effect sizes):

$$\mathbf{e}_{g} = \mathbf{X}_{g}\mathbf{w} + \boldsymbol{\epsilon}_{g},\ \ \boldsymbol{\epsilon}_{g} \sim \mathrm{N}(0, \sigma_{\epsilon}^{2}\mathbf{I}), \qquad \text{(Equation S1)}$$

based on summary-level reference data that contain marginal eQTL effect sizes ($\widetilde{w}_j, j = 1, \dots, m$) and p-values from the following single variant regression model:

$$\mathbf{e}_{g} = \mathbf{X}_{j}w_{j} + \boldsymbol{\epsilon}_{j},\ \ \boldsymbol{\epsilon}_{j} \sim \mathrm{N}(0, \sigma_{\epsilon_j}^{2}\mathbf{I}),\ j\ =\ 1, \dots, m. \qquad \text{(Equation S2)}$$

Here, $\mathbf{e}_g$ is a vector representing gene expression levels of gene g, $\mathbf{X}_j$ is an $n \times 1$ vector of genotype data for genetic variant j, $\mathbf{X}_{\mathbf{g}}$ is an $n \times m$ matrix of genotype data of SNP predictors proximal or within gene g, $\mathbf{w}$ is the eQTL effect-size vector, n is the sample size, and $\boldsymbol{\epsilon}_j$ and $\boldsymbol{\epsilon}_g$ are the error terms.

Assuming $\mathbf{e}_{\mathbf{g}}$ and the column of $\mathbf{X}_{\mathbf{g}}$ to be standardized, $\mathbf{e}_{g}^{T}\mathbf{e}_{g} = n$, we can show that the marginal least squared eQTL effect size estimates from Equation S2 is $\widetilde{\mathbf{w}} = \mathbf{X}_{\mathbf{g}}^{T}\mathbf{e}_{g}/n$ and that the LD correlation matrix is $\mathbf{R} = \mathbf{X}_{\mathbf{g}}^{T}\mathbf{X}_{\mathbf{g}}/n$. OTTERS uses four summary-data based polygenic risk score (PRS) methods in Stage I — P-value Thresholding with linkage disequilibrium (LD) clumping (P+T)[1], frequentist LASSO[2] regression based method lassosum[3], Bayesian Dirichlet Process Regression model[4] based method SDPR[5], and Bayesian multivariate regression model based method PRS-CS[6]. The P+T method is described in the main text. Here, we describe details of the other three methods.

3.1.1 Frequentist lassosum

The LASSO[2] estimates $\hat{\mathbf{w}}$ by minimizing the following loss function with a penalty parameter λ,

$$f(\mathbf{w}) = \frac{\mathbf{e}_g^T\mathbf{e}_g}{n} + \mathbf{w}^T\mathbf{R}\mathbf{w} - \mathbf{2}\mathbf{w}^T\widetilde{\mathbf{w}} + 2\lambda\,\|\mathbf{w}\|_1. \qquad \text{(Equation S3)}$$

Since $\mathbf{R}$ is generally not available in eQTL summary data, $\mathbf{R}$ would be approximated by the LD correlation matrix ($\mathbf{R}_r$) from an external reference panel such as one from the 1000 Genomes Project[7]. Because $\mathbf{R_r}$ and $\widetilde{\mathbf{w}}$ are derived from genotype data of different samples, the standard LASSO estimates will be unstable and non-unique[3]. The frequentist lassosum[3] method replaces $\mathbf{R}$ by $\mathbf{R}_s = (1-s)\mathbf{R_r} + s\mathbf{I}$ in Equation S3 with a tuning parameter $0 < s < 1$ and an identity matrix $\mathbf{I}$, and then estimates $\widehat{\mathbf{w}}$ by minimizing the following loss function:

$$f(\mathbf{w}) = \frac{\mathbf{e}_g^T\mathbf{e}_g}{n} + (1-s)\,\mathbf{w}^T\mathbf{R_r}\mathbf{w} - 2\mathbf{w}^T\widetilde{\mathbf{w}} + s\mathbf{w}^T\mathbf{w} + 2\lambda\|\mathbf{w}\|_1. \quad \text{(Equation S4)}$$

Let $\mathbf{X}_r$ denote reference genotype data that are different from the genotype data used to estimate $\widetilde{\mathbf{w}}$, and $\mathbf{R}_r = \mathbf{X}_r^T\mathbf{X}_r/n_r$ denote the reference LD correlation matrix with reference sample size n_r. Given s and λ, the eQTL effect sizes $\widehat{\mathbf{w}}$ minimizing $f(\mathbf{w})$ in Equation S4 can be obtained by iteratively updating $\widehat{w}_j$ as

$$\widehat{w}_j^{(k)} = \begin{cases} \dfrac{\operatorname{sign}\left(\mu_j^{(k)}\right)\left|\mu_j^{(k)} - \lambda\right|}{(\widetilde{\mathbf{X}}_j^T\widetilde{\mathbf{X}}_j + s)} & \left|\mu_j^{(k)}\right| - \lambda > 0 \\ 0 & \text{otherwise} \end{cases}$$

$$\mu_j^{(k)} = \widetilde{w}_j - \widetilde{\mathbf{X}}_j^T\left(\widetilde{\mathbf{X}}\widehat{\mathbf{w}}^{(k-1)} - \widetilde{\mathbf{X}}_j\widehat{w}_j^{(k-1)}\right)$$

where k and $k-1$ are the iteration numbers; $\widetilde{\mathbf{X}} = \sqrt{1-s}\,\mathbf{X}_r$; and $\widetilde{\mathbf{X}}_j$ is the genotype data vector for genetic variant j in $\widetilde{\mathbf{X}}$.

To select suitable values for tuning parameters s and λ, the lassosum method uses a pseudovalidation procedure that can approximate the correlation between the imputed $\widehat{\mathbf{GReX}}$ and the gene expression in the test data using a shrunken estimate of $\widetilde{\mathbf{w}}$. In our simulation and application studies, pseudovalidation was used to tune λ from a grid of 20 equally spaced values from 0.001 to 0.1 on a log-scale and s from (0.2, 0.5, 0.9, 1). We used the lassosum package (https://github.com/tshmak/lassosum) to implement the iterative optimization and the

pseudovalidation procedure. The corresponding proof of iterative optimization and the detailed information of the pseudovalidation procedure can be found in the Supplementary materials and the Material and methods section of the lassosum paper[3].

3.1.2 Nonparametric Bayesian DPR Method SDPR

Following the idea of nonparametric Bayesian DPR model proposed in previous studies[4], the prior of w_j is assumed to be a normal distribution with mean 0 and variance σ_w^2, with the prior of σ_w^2 assumed to be a Dirichlet process prior[8]:

$$w_j \sim N(0, \sigma_w^2),\ \sigma_w^2 \sim DP(H, \alpha);$$

where the distribution on σ_w^2 deviates from the DP with the base distribution H and the concentration parameter α controls the shrinkage of the distribution on σ_w^2 towards H. An equivalent DP normal mixture model can be derived by viewing σ_w^2 as a latent variable that can be integrated out:

$$w_j|\pi_j, \sigma_j^2 \sim \sum_{j=0}^{+\infty} \pi_j\, N\left(0, \sigma_j^2\right), \qquad \pi_j = v_j \prod_{l=0}^{j-1} (1 - v_l)\,, \qquad \sigma_j^2 \sim H,$$

$$v_l \sim Beta(1, \alpha),\quad l\ =\ 0, \cdots, j-1, j;$$

The resulting Gaussian mixture prior of w_j is a weighted sum of an infinite number of normal distributions $N(0, \sigma_j^2)$ with weights π_j determined by v_l with a Beta prior. The concentration parameter α in the Beta prior distribution determines the number of components with non-zero weights in the mixture normal prior. This infinite Gaussian mixture prior can approximate a wide range of continuous parametric prior distributions assumed by other PRS methods[5,9], for example, the normal distribution in LDpred[10], the point normal mixture distribution in LDpred[10] and LDpred2[11], and the three-point normal mixture distribution in SBayesR[12].

SDPR[5] implemented DPR but can be applied to summary statistics (i.e., marginal eQTL effect sizes $\widetilde{\mathbf{w}}$) as the input by assuming $\widetilde{\mathbf{w}}|\mathbf{w} \sim N\left(\mathbf{Rw}, \frac{\mathbf{R}+na\mathbf{I}}{n}\right)$, where a is a constant to shrink

the covariance between two eQTLs, $\mathbf{R}$ is the LD correlation matrix of test eQTLs, and n is the training sample size. The SDPR method overparameterizes the model by writing $\mathrm{w_j} = \eta\gamma_\mathrm{j}$ and use the square of uniform distribution as the base distribution H, following Gelman's advice[13]. The SDPR method implements a parallel Markov Chain Monte Carlo (MCMC) algorithm in C++ to obtain posterior estimates of η and γ_j (i.e., eQTL effect sizes $\widehat{\mathbf{w}} = \hat{\eta}\widehat{\boldsymbol{\gamma}}$), conditioning on marginal eQTL effect sizes $\widetilde{\mathbf{w}}$ and reference LD correlation matrix $\mathbf{R}$. The SDPR software also designs an algorithm for partitioning approximately independent LD blocks to ensure that all SNPs in one LD block have no nonignorable correlation with SNPs in other blocks. More details about the MCMC Algorithm and the partition of the reference LD matrix can be found in the Supplemental Text of the SDPR paper[5]. For all the parameters, we used the default setting in the SDPR software (https://github.com/eldronzhou/SDPR) for our simulation and real studies.

3.1.3 Bayesian Multivariate Regression Method PRS-CS

Following the continuous shrinkage prior proposed by PRS-CS[6], we assume the following normal prior for $\mathrm{w_j}$ and non-informative scale-invariant Jeffreys prior on the residual variance σ_ϵ^2 in the Equation S1:

$$\mathrm{w_j} \sim \mathrm{N}\left(0, \frac{\sigma_\epsilon^2}{\mathrm{n}}\psi_\mathrm{j}\right), \quad \mathrm{p}(\sigma_\epsilon^2) \propto \sigma_\epsilon^2; \quad \psi_\mathrm{j} \sim \mathrm{Gamma}(\mathrm{a}, \delta_\mathrm{j}), \delta_\mathrm{j} \sim \mathrm{Gamma}(\mathrm{b}, \phi),$$

where local shrinkage parameter ψ_j has an independent gamma-gamma prior and ϕ is a global-shrinkage parameter controlling the overall sparsity of $\mathbf{w}$. Posterior estimates of the eQTL effect sizes $\widehat{\mathbf{w}}$ will be obtained by using only summary-level eQTL data $\widetilde{\mathbf{w}}$ and the reference LD correlation matrix $\mathbf{R}$ by Gibbs Sampler:

$$\mathbf{w} \mid \sigma_\epsilon^2, \boldsymbol{\Psi}, \widetilde{\mathbf{w}}, \mathbf{R} \sim \mathrm{MVN}\left(\frac{\mathrm{n}}{\sigma_\epsilon^2}\boldsymbol{\Sigma}\widetilde{\mathbf{w}}, \boldsymbol{\Sigma}\right), \boldsymbol{\Sigma} = \frac{\sigma_\epsilon^2}{\mathrm{n}}(\mathbf{R} + \boldsymbol{\Psi}^{-1})^{-1}$$

where $\boldsymbol{\Psi} = \mathrm{diag}\{\psi_1, \psi_2, \cdots, \psi_\mathrm{m}\}$. Detailed Gibbs updates of σ_ϵ^2, ψ_j, and δ_j in each MCMC iteration has been provided in the Supplemental Note of the PRS-CS paper[6].

PRS-CS provides two algorithms with different strategies to learn the global shrinkage parameter ϕ. One is to place a half-Cauchy prior on ϕ with $\phi^{1/2} \sim \mathrm{C}^{+}(0,1)$, and perform a fully Bayesian approach to learn ϕ from the data, which is referred as PRS-CS auto. Another is to search a small number of fixed ϕ and select the value producing the best predictive performance in the validation data set. In the work, we applied PRS-CS with fixed ϕ, because PRS-CS auto tended to fail when the proportion of causal variants is small and the performance of PRS-CS is not sensitive to the choice of the fixed ϕ[6]. Following the suggestion from the PRS-CS paper, we set ϕ as the square of the proportion of causal variants in the simulation studies and as 10^{-4} for each gene in the real data applications. For other parameters, we used the default setting in the PRS-CS software (https://github.com/getian107/PRScs).

3.2 OTTERS Stage II Gene-based Association Test

Once we train eQTL weights by using each training method from the summary-level reference eQTL data, we will perform the respective gene-based association analysis for that method by taking the trained eQTL weights as corresponding SNP weights. We thus derive a set of TWAS p-values per gene g; one p-value for each training method that we applied. Finally, we will employ the aggregated Cauchy association test (ACAT-O) approach[14] to combine the TWAS p-values from individual training methods and conduct an omnibus test. We refer to these p-values obtained by ACAT-O tests as OTTERS p-values. We describe how we perform the gene-based association analysis and construct the OTTERS p-values below.

3.2.1 Gene-based Association Test with eQTL Weights

Given individual-level GWAS data with genotype $\mathbf{X}_{\text{new}}$ and phenotype $\mathbf{y}$, the GReX for $\mathbf{X}_{\text{new}}$ can be imputed by $\widehat{\mathbf{GReX}} = \mathbf{X}_{\text{new}}\, \hat{\mathbf{w}}$. The follow-up TWAS using a gene-based burden

testing is to assess the association between $\widehat{\mathbf{GReX}}$ and $\mathbf{y}$ based on the following generalized linear regression model:

$$f(E[\mathbf{y}|\mathbf{X_{new}}, \mathbf{C}] = \theta\mathbf{C} + \beta\,\widehat{\mathbf{GReX}},$$

where $\mathbf{C}$ denotes the matrix for covariates associated with $\mathbf{y}$, and $f(\cdot)$ denotes a pre-specified link function. This link function can be set as identity function for quantitative phenotype and logit function for dichotomous phenotype. The gene-based association test is equivalent to test $H_0: \beta = 0$.

When summary-level GWAS data are available, one can use the TWAS Z-score test statistics as used by FUSION[15] and S-PrediXcan[16] as follows:

$$Z_{g,FUSION} = \frac{\sum_{j=1}^{J}(\widehat{w}_j Z_j)}{\sqrt{\widehat{\boldsymbol{w}}'\boldsymbol{V}\widehat{\boldsymbol{w}}}}, \quad Z_{g,S-PrediXcan} = \frac{\sum_{j=1}^{J}(\widehat{w}_j \widehat{\sigma}_j Z_j)}{\sqrt{\widehat{\boldsymbol{w}}'\boldsymbol{V}\widehat{\boldsymbol{w}}}};$$

where Z_j is the single variant Z-score test statistic in GWAS for the jth SNP, $j = 1, \dots, J$, for all test SNPs that have both eQTL weights with respect to the test gene g and GWAS Z-scores; $\widehat{\sigma}_j$ is the genotype standard deviation of the jth SNP; and $\mathbf{V}$ denotes the genotype correlation matrix in FUSION Z-score statistic and genotype covariance matrix in S-PrediXcan Z-score statistic of the test SNPs.

The Supplementary Text of our previous work TIGAR-V2[17] has proved that when $\widehat{\mathbf{w}}$ are estimated using standardized reference data, FUSION Z-score statistic is equivalent to the SPrediXcan test statistic; while when $\widehat{\mathbf{w}}$ are estimated using non-standardized reference data, FUSION Z-score statistic will lead to inflated Type I error and SPrediXcan test statistic should be used in this situation. In this work, we obtained standardized $\widehat{\mathbf{w}}$ from the lassosum, SDPR, and PRS-CS software. For P+T, we estimated the standardized effect size based on the Z-score statistic values in the eQTL summary data by $\widetilde{w}_j \approx Z_j/\sqrt{\mathrm{median}(n_{g,j})}$, where $\mathrm{median}(n_{g,j})$ denotes the median sample size of all cis-eQTLs for the target gene g.

3.2.2 OTTERS p-value by ACAT-O Test

Following the idea of ACAT-O[14] to combine the TWAS p-values using eQTL weights trained by different methods from the summary-level reference eQTL data, we create our OTTERS test statistics as:

$$T_{OTTERS} = \frac{1}{K}\sum_{k=1}^{K} \tan\{(0.5 - p_k)\,\pi\}, \qquad \pi \approx 3.14;$$

where p_k is the TWAS p-value with eQTL weights trained using the kth method, e.g., P+T(0.001), P+T(0.05), lassosum, SDPR, and PRS-CS (K=5). Under null hypothesis, the p-value p_k is uniformly distributed in $[0, 1]$ and the transformation $\tan\{(0.5 - p_k)\,\pi\}$ is Cauchy distributed. Thus, the null distribution of the test statistic T_{OTTERS} can be approximated by a Cauchy distribution[18] and the corresponding OTTERS p-value (p_{OTTERS}) can be approximated by

$$p_{OTTERS} \approx \frac{1}{2} - \frac{\{\arctan(T_{OTTERS})\}}{\pi}.$$

Supplemental References

1. Purcell, S.M., Wray, N.R., Stone, J.L., Visscher, P.M., O'Donovan, M.C., Sullivan, P.F., Sklar, P., Purcell (Leader), S.M., Stone, J.L., Sullivan, P.F., et al. (2009). Common polygenic variation contributes to risk of schizophrenia and bipolar disorder. Nature 460, 748–752.

2. Tibshirani, R. (1996). Regression Shrinkage and Selection Via the Lasso. Journal of the Royal Statistical Society: Series B (Methodological) 58, 267–288.

3. Mak, T.S.H., Porsch, R.M., Choi, S.W., Zhou, X., and Sham, P.C. (2017). Polygenic scores via penalized regression on summary statistics. Genetic Epidemiology 41, 469–480.

4. Zeng, P., and Zhou, X. (2017). Non-parametric genetic prediction of complex traits with latent Dirichlet process regression models. Nat Commun 8, 456.

5. Zhou, G., and Zhao, H. (2021). A fast and robust Bayesian nonparametric method for prediction of complex traits using summary statistics. PLoS Genet 17, e1009697.

6. Ge, T., Chen, C.-Y., Ni, Y., Feng, Y.-C.A., and Smoller, J.W. (2019). Polygenic prediction via Bayesian regression and continuous shrinkage priors. Nat Commun 10, 1776.

7. Auton, A., Abecasis, G.R., Altshuler, D.M., Durbin, R.M., Abecasis, G.R., Bentley, D.R., Chakravarti, A., Clark, A.G., Donnelly, P., Eichler, E.E., et al. (2015). A global reference for human genetic variation. Nature 526, 68–74.

8. Müller, P., and Mitra, R. (2013). Bayesian Nonparametric Inference – Why and How. Bayesian Anal 8, 10.1214/13-BA811.

9. Lijoi, A., Prünster, I., and Walker, S.G. (2005). On Consistency of Nonparametric Normal Mixtures for Bayesian Density Estimation. Journal of the American Statistical Association 100, 1292–1296.

10. Vilhjálmsson, B.J., Yang, J., Finucane, H.K., Gusev, A., Lindström, S., Ripke, S., Genovese, G., Loh, P.-R., Bhatia, G., Do, R., et al. (2015). Modeling Linkage Disequilibrium Increases Accuracy of Polygenic Risk Scores. Am. J. Hum. Genet. 97, 576–592.

11. Privé, F., Arbel, J., and Vilhjálmsson, B.J. (2020). LDpred2: better, faster, stronger. Bioinformatics 36, 5424–5431.

12. Lloyd-Jones, L.R., Zeng, J., Sidorenko, J., Yengo, L., Moser, G., Kemper, K.E., Wang, H., Zheng, Z., Magi, R., Esko, T., et al. (2019). Improved polygenic prediction by Bayesian multiple regression on summary statistics. Nat Commun 10, 5086.

13. Gelman, A. (2006). Prior distributions for variance parameters in hierarchical models (comment on article by Browne and Draper). Bayesian Analysis 1, 515–534.

14. Liu, Y., Chen, S., Li, Z., Morrison, A.C., Boerwinkle, E., and Lin, X. (2019). ACAT: A Fast and Powerful p Value Combination Method for Rare-Variant Analysis in Sequencing Studies. The American Journal of Human Genetics 104, 410–421.

15. Gusev, A., Ko, A., Shi, H., Bhatia, G., Chung, W., Penninx, B.W.J.H., Jansen, R., de Geus, E.J.C., Boomsma, D.I., Wright, F.A., et al. (2016). Integrative approaches for large-scale transcriptome-wide association studies. Nat Genet 48, 245–252.

16. Barbeira, A.N., Dickinson, S.P., Bonazzola, R., Zheng, J., Wheeler, H.E., Torres, J.M., Torstenson, E.S., Shah, K.P., Garcia, T., Edwards, T.L., et al. (2018). Exploring the phenotypic consequences of tissue specific gene expression variation inferred from GWAS summary statistics. Nature Communications 9, 1825.

17. Parrish, R.L., Gibson, G.C., Epstein, M.P., and Yang, J. (2022). TIGAR-V2: Efficient TWAS tool with nonparametric Bayesian eQTL weights of 49 tissue types from GTEx V8. Human Genetics and Genomics Advances 3, 100068.

18. Liu, Y., and Xie, J. (2020). Cauchy Combination Test: A Powerful Test With Analytic p-Value Calculation Under Arbitrary Dependency Structures. Journal of the American Statistical Association 115, 393–402.
