# Supplementary material for: OTTERS: a powerful TWAS framework leveraging summary-level reference data
Source: Nat Commun. 2023 Mar 7;14:1271. doi: 10.1038/s41467-023-36862-w (PMC9992663; doi:10.1038/s41467-023-36862-w)
Supplement: Supplementary file 3 — Reporting Summary [file 41467_2023_36862_MOESM3_ESM.pdf]

nature portfolio

Corresponding author(s): Michael P. Esptein; Jingjing Yang

Last updated by author(s): Jan 28, 2023

Reporting Summary

Nature Portfolio wishes to improve the reproducibility of the work that we publish. This form provides structure for consistency and transparency in reporting. For further information on Nature Portfolio policies, see our Editorial Policies and the Editorial Policy Checklist.

Statistics

For all statistical analyses, confirm that the following items are present in the figure legend, table legend, main text, or Methods section.

n/a	Confirmed	
☐	☒	The exact sample size (*n*) for each experimental group/condition, given as a discrete number and unit of measurement
☒	☐	A statement on whether measurements were taken from distinct samples or whether the same sample was measured repeatedly
☐	☒	The statistical test(s) used AND whether they are one- or two-sided<br>*Only common tests should be described solely by name; describe more complex techniques in the Methods section.*
☐	☒	A description of all covariates tested
☐	☒	A description of any assumptions or corrections, such as tests of normality and adjustment for multiple comparisons
☐	☒	A full description of the statistical parameters including central tendency (e.g. means) or other basic estimates (e.g. regression coefficient) AND variation (e.g. standard deviation) or associated estimates of uncertainty (e.g. confidence intervals)
☐	☒	For null hypothesis testing, the test statistic (e.g. *F*, *t*, *r*) with confidence intervals, effect sizes, degrees of freedom and *P* value noted<br>*Give P values as exact values whenever suitable.*
☐	☒	For Bayesian analysis, information on the choice of priors and Markov chain Monte Carlo settings
☒	☐	For hierarchical and complex designs, identification of the appropriate level for tests and full reporting of outcomes
☐	☒	Estimates of effect sizes (e.g. Cohen's *d*, Pearson's *r*), indicating how they were calculated

Our web collection on statistics for biologists contains articles on many of the points above.

Software and code

Policy information about availability of computer code

Data collection	All data used in this study were downloaded from public repository or dbGap. No new data were collected in this study. Please see Data Availability section for details.
Data analysis	Source code for OTTERS is available from https://github.com/daiqile96/OTTERS. All scripts used to generate intermediate or final data and figures are available from github page https://github.com/daiqile96/OTTERS_paper64. Source code for ACAT is available from https:// github.com/yaowuliu/ACAT. Source code for FUSION is available from http://gusevlab.org/projects/fusion. Source code for lassosum is available from https://github.com/tshmak/lassosum. Source code for PRS-CS is available from https://github.com/getian107/PRScs. Source code for SDPR is available from https://github.com/eldronzhou/SDPR. Plink version 1.9 is used and available at https://www.cog-genomics.org/plink/.

For manuscripts utilizing custom algorithms or software that are central to the research but not yet described in published literature, software must be made available to editors and reviewers. We strongly encourage code deposition in a community repository (e.g. GitHub). See the Nature Portfolio guidelines for submitting code & software for further information.

Data

Policy information about availability of data

All manuscripts must include a data availability statement. This statement should provide the following information, where applicable:

- Accession codes, unique identifiers, or web links for publicly available datasets
- A description of any restrictions on data availability
- For clinical datasets or third party data, please ensure that the statement adheres to our policy

ROS/MAP/MSBB WGS data used in our simulation studies are available through Synapse with data access application (https://www.synapse.org/#!Synapse:syn10901595). The eQTLGen consortium data are available from the consortium portal website (https://www.eqtlgen.org). UK Biobank summary-level GWAS data are available through the Alkes Group (https://alkesgroup.broadinstitute.org/UKBB). Individual-level GTEx reference data are available through dbGap (Accession phs000424.v8.p2). Summary eQTL data of blood tissue in GTEx cohort are available from GTEx Portal (https://console.cloud.google.com/storage/browser/gtex-resources/GTEx_Analysis_v8_QTLs/GTEx_Analysis_v8_eQTL_all_associations). Significant genes from TWAS-hub are available from http://twas-hub.org. The summary eQTL weights of blood tissue generated by OTTERS (from eQTLGen data) and summary TWAS results generated by OTTERS for cardiovascular disease (from UK Biobank data) are available from Synapse (https://doi.org/10.7303/syn51009573).

Human research participants

Policy information about studies involving human research participants and Sex and Gender in Research.

Reporting on sex and gender	Not relevant in this study.
Population characteristics	All data used in this study are summary level data and de-identified genetics data of European population.
Recruitment	N/A
Ethics oversight	All data used in this study are de-identified genotype data and summary level eQTL and summary level GWAS data. ROS/MAP genotype data were collected with ethics approval from the IRB at Rush University and all participants consented to participate. Only summary level GWAS data of UK Biobank were used in this study. No IRB approval is needed for analyzing publicly available summary level eQTL and GWAS data, per requirement by NIH.

Note that full information on the approval of the study protocol must also be provided in the manuscript.

Field-specific reporting

Please select the one below that is the best fit for your research. If you are not sure, read the appropriate sections before making your selection.

☒ Life sciences ☐ Behavioural & social sciences ☐ Ecological, evolutionary & environmental sciences

For a reference copy of the document with all sections, see nature.com/documents/nr-reporting-summary-flat.pdf

Life sciences study design

All studies must disclose on these points even when the disclosure is negative.

Sample size	Sample size was referred to the number of individuals whose whole blood tissue were used to profile RNA data in the eQTL summary data. Sample size was referred to the number of individuals whose genetic data were profiled by UK Biobank in the GWAS summary data. This study dose not collect any new data. No statistical methods were used to determine sample size. Power analysis is not relevant in this study. Sample size in the simulation data were chosen to show appropriate power around 80%.
Data exclusions	Genes with training $R2$ of genetically regulated gene expression component <0.01 were excluded in the UK Biobank data analysis, because low training $R2$ means the genetically regulated gene expression component is too low to explain possible mediation TWAS effect.
Replication	Our simulation studies demonstrated the power and type I error of our method. The TWAS results by another widely used TWAS tool FUSION partially validated our results.
Randomization	Randomization is rigorously implemented in our simulation studies. All samples in the real data were randomly collected from a population.
Blinding	This study is proposing a analytical method/tool to leverage publicly available summary level eQTL and GWAS data. No experiments were conducted in this study, thus blinding is not directly relevant.

Reporting for specific materials, systems and methods

We require information from authors about some types of materials, experimental systems and methods used in many studies. Here, indicate whether each material, system or method listed is relevant to your study. If you are not sure if a list item applies to your research, read the appropriate section before selecting a response.

Materials & experimental systems

n/a	Involved in the study
☒	☐ Antibodies
☒	☐ Eukaryotic cell lines
☒	☐ Palaeontology and archaeology
☒	☐ Animals and other organisms
☒	☐ Clinical data
☒	☐ Dual use research of concern

Methods

n/a	Involved in the study
☒	☐ ChIP-seq
☒	☐ Flow cytometry
☒	☐ MRI-based neuroimaging
